# Implementing a weight-specific quality-of-life tool for young people in primary health care: a qualitative study

**DOI:** 10.3399/BJGPO.2021.0052

**Published:** 2021-06-30

**Authors:** Yemi Oluboyede, Sarah R Hill, Suzanne McDonald, Emily Henderson

**Affiliations:** 1 Population Health Science Institute, Baddiley-Clark Building, Richardson Road, Newcastle University, Newcastle upon Tyne, UK; 2 Centre for Clinical Research, The University of Queensland, Royal Brisbane & Women’s Hospital Campus, Herston, Queensland, Australia; 3 Children & Young People’s Mental Health & Wellbeing, Social Work, Education & Community Wellbeing, Northumbria University, Newcastle-upon-Tyne, UK

**Keywords:** paediatric obesity, quality of life, primary health care, attitude, interview, consultation

## Abstract

**Background:**

Obesity is thought to be one of the most serious global public health challenges of the 21st century. The primary care setting is important in terms of the diagnosis, education, and management of obesity in children and young people. This study explored the views of primary care clinicians on the implementation of a quality-of-life (QoL) tool to help young people and their families identify the impact of weight on QoL.

**Aim:**

To assess the acceptability and feasibility of implementing the Weight-specific Adolescent Instrument for Economic-evaluation (WAItE) QoL tool for young people aged 11–18 years in primary care.

**Design & setting:**

Qualitative study in Northern England, UK

**Method:**

One-to-one, semi-structured interviews were conducted with a purposive sample of primary healthcare clinicians working in practices located in areas of varying deprivation in Northern England, UK. Interview transcripts were coded and analysed using framework analysis in NVivo (version 10).

**Results:**

Participants (*n* = 16 GPs; *n* = 4 practice nurses) found the WAItE tool acceptable for them and their patients, and believed it was feasible for use in routine clinical practice. It was important to primary care clinicians that the tool would provide an overall QoL score that would be easy for GPs and nurses to interpret, to help them identify patients most in need of specialist help.

**Conclusion:**

This study has developed a platform for further research around QoL in young people who are overweight and obese. A future feasibility study will focus on implementing the tool in a small number of primary healthcare practices.

## How this fits in

Being overweight or obese can negatively impact an individual’s QoL, yet clinicians and patients find it difficult to discuss overweight and obesity. Focusing weight conversations on QoL may help refocus sensitive conversations. Limited evidence has explored the use of weight-specific QoL tools in primary health care, particularly with young people. This study explored the views of primary healthcare professionals about the use of the WAItE tool for discussing QoL with young people who are overweight and obese.

## Introduction

Childhood obesity is one of the most serious global public health challenges of the 21st century, with an estimated 381 million people aged 0–19 years measured as overweight or obese by body mass index (BMI).^1^ Young people who are overweight and obese are more likely to be overweight or obese in adulthood, and have a higher risk of illness, disability, and dying earlier.^2^ These young people are also more likely to experience low QoL, owing to associated physical and mental health comorbidities, which can often extend into adulthood.^3^ Primary care could play a pivotal role in addressing the needs of young people and families.

Puhl and Heuer’s^4^ literature review shows that the negative consequences of weight stigma affect one’s mental health (for example, depression, low self-esteem, poor body image, and psychiatric disorders) as well as one’s physical health (for example, maladaptive eating behaviours, exercise avoidance, and reduced motivation to lose weight). Information on QoL may also influence parental motivation to change behaviours and improve adherence with recommendations for behaviour change. Clinicians and patients find discussing obesity difficult for a range of reasons, namely owing to the sensitive nature of the topic and the very few effective treatment options available.^5^

Tools to refocus sensitive conversations about weight on other health outcomes associated with weight, rather than the body itself, may aid dialogue. One example is Public Health England’s step-by-step guide for primary care professionals to carry out conversations around weight with families of children and young people.^6^ However, this approach is limited given that the guide focuses only on supporting the primary care professional as opposed to including young people and their families. Focusing discussion on the QoL of young people may help to complement this guide. The WAItE is a validated QoL tool that was developed from young people’s views on aspects of their lives affected by weight — tiredness, walking, participation in sports, concentration, embarrassment, unhappiness, and being treated differently — and can be used to measure the impact of overweight and obesity on QoL.^7–9^ This study aimed to explore the views of primary care clinicians about the feasibility and acceptability of administering the WAItE tool in clinical practice.

## Method

### Design

One-to-one, semi-structured interviews were conducted with a purposive sample of primary care clinicians to elicit their views about different aspects related to the feasibility and acceptability of implementing the WAItE tool in routine clinical practice.

### Participants

Participants were recruited from primary healthcare practices in northern England between June 2017 and December 2017. Relevant practices were identified using Public Health England’s National General Practice Profiles,^10^ according to clinical commissioning group location (groups of GPs who commission services for their local area).^11^ The sample was stratified by percentage of patients aged <18 years and percentage classified as obese based on BMI (monitored by each practice according to the obesity Quality and Outcomes Framework [QOF]).^12^ To ensure sample diversity, the deprivation scores of each GP practice location and the percentage of patients who were of working status were also used to stratify GP practices. Practices were recruited via an email to the primary healthcare practice manager inviting GPs and practice nurses to participate. Participants were also recruited via the Clinical Research Network, which distributed invitations to primary healthcare practices registered as research active.

### Interview schedule

The interview schedule covered four topic areas developed by the research team drawing on key topics identified in the literature. Questions and prompts for each topic area assessed practitioner views on their: 1) experience working with young people who are overweight or obese; 2) beliefs about the link between weight and QoL in young people; 3) attitudes towards using QoL tools in primary health care; and 4) views about the feasibility of using the WAItE tool in their clinical practice. The interview schedule was piloted with three healthcare clinicians for comprehension and underwent refinements accordingly.

### Interview procedure

Participants were interviewed once at their work premises, own homes, or via telephone, according to their preferences. Interviews were conducted by two female researchers, both of whom are experienced qualitative researchers and qualified at PhD level. Interviews were audio-recorded and field notes were taken. Participants were provided with a paper copy of the WAItE tool to examine during the interview (a copy of the WAItE tool is available from the authors on request).

### Data analysis

Interviews were transcribed verbatim and anonymised by a professional transcriber, and uploaded into NVivo software (version 10) to facilitate analysis. Framework analysis was conducted using a five-step standard procedure.^13^

In step one, the first three interview transcripts were selected for review, and key ideas and recurrent themes from each transcript were noted. In step two, a thematic framework was developed using the ideas and themes identified in step one and the four distinct topic areas in the interview schedule. In step three, the whole dataset was analysed by applying the thematic framework created in step two. The thematic framework underwent refinements over time in order to respond to emergent and analytical themes. During this process, judgments were made about meaning, relevance, and importance of each statement in relation to the interview as a whole, and with particular emphasis on common and divergent themes in the data. A standard procedure was used to assess and confirm data saturation (that is, the point at which no new themes emerge from the data) during this stage.^14^ In step four, data within each theme were arranged in thematic charts, which allowed systematic comparisons between the views of subgroups of interest including clinician (GP or practice nurse), sex, and number of years working in clinical practice. In step five, associations, patterns, and emergent themes were systematically identified from the data.

The analysis plan was developed and monitored in regular discussions by the research team. An initial dataset was coded independently by one researcher (SM), and a subset of data was discussed with other members of the research team (YO, EH). The purpose was to cross-check interpretations of key themes emerging from the data and jointly agree the final coding frame to be applied in the analysis of the remaining data.

### Ethical considerations

All participants provided informed, written consent before participation and received a £10 gift voucher after completing the interview. General Data Protection Regulation (GDPR) was followed for the collection, storage, and use of data. This study adhered to the Standards for Reporting Qualitative Research^15^ and the consolidated criteria for reporting qualitative research (see Supplementary Appendix S1).^16^

## Results

Twenty participants (*n* = 15 female, *n* = 5 male) were interviewed, four of whom were practice nurses and the remaining participants were GPs. The interviews lasted between 19 and 81 minutes (see Table 1). The percentage of patients aged <18 years registered to the participants’ primary healthcare practice ranged from 12.4–27.9% (England average: 20.7%), and the QOF reported obesity prevalence for both adults and children in each practice ranged from 6.81–18.12% (England average: 9.5%). Table 1 displays further details about the characteristics of participants and their practices.

**Table 1.** Participant characteristics

ID	Approximate length of interview, minutes	Sex	Clinician	GP practice deprivation score(England 21.8)	% aged <18 years in GP area(England: 20.7%)	% unemployed in GP area(England: 4.4%)	% obesity: QOF prevalence(England: 9.5%)
1	36:07	F	GP	7.9	27.9	3.0	8.65
2	53:01	F	GP	50.8	26.5	15.4	12.07
3	21:37	F	GP	22.8	19.5	15.0	11.74
4	19:23	F	GP	34.7	14.5	7.1	9.28
5	44:19	M	GP	28.6	18.7	3.0	13.12
6	20:48	M	GP	34.7	14.5	7.1	9.28
7	21:50	F	GP	22.8	19.5	15.0	11.74
8	33:46	F	Practice nurse	34.8	20.8	11.8	18.12
9	36:18	M	GP	9.0	18.1	2.3	8.21
10	27:30	F	GP	16.3	20.6	7.6	9.95
11	26:33	F	GP	17.7	22.6	10.6	14.43
12	18:29	F	GP	13.2	17.7	0.9	7.81
13	21:16	M	GP	31.7	18.9	0.9	14.81
14	31:26	F	Practice nurse	31.7	18.9	0.9	14.81
15	52:42	F	GP	15.7	15.8	0.8	11.86
16	1:21:51	M	GP	13.3	16.6	4.8	13.13
17	44:48	F	GP	26.0	12.4	0.0	6.81
18	35:04	F	Practice nurse	15.4	16.7	0.9	10.47
19	29:02	F	GP (locum)	48.4	20.0	14.2	13.40
20	38:53	F	Practice nurse	16.3	20.6	7.6	9.95

QOF = Quality and Outcomes Framework.

Key themes emerged within each of the four topic areas from the interview schedule; however, it is beyond the scope of this article to discuss emergent themes from all topic areas. The most salient emergent themes for addressing primary care clinicians’ views on the feasibility and acceptability of administering the WAItE tool in clinical practice are presented below. Themes were inclusive of all data and, therefore, represent the views of all participants. Divergent views within each theme are highlighted where they occurred. Supporting quotes for each theme are presented in Tables 2–4.

**Table 2.** Supporting quotes for the 'Views about QoL tools in clinical practice' theme

**Sub-theme**	**Example quotes**
Clinicians’ experiences using QoL tools in clinical practice	*'I suppose I do use questionnaires. I use mood questionnaires for patients who are depressed or that sort of thing, use that sort of thing … But not for people who are overweight.'* (P1, Female, GP)*'I haven’t really used them, to be honest. I’m trying to think. The only thing … doing the questionnaire or anything I’ve not used specific quality-of-life tools. The only questionnaire thing I used to routinely give to patients is a PHQ9 about depression, but I don’t routinely use quality-of-life tools.'* (P11, Female, GP)*'I can’t remember using a quality-of-life tool. I know some exist so there’s the rheumatoid arthritis quality-of-life tool … What I’ve always preferred to use is just general talk and intuition.'* (P16, Male, GP)
Perceived advantages of QoL tools	*'It will bring up a subject, doesn’t it, so it allows you to explore that subject a little bit more, and thinking about quality of life and maybe how we can improve it.'* (P8, Female, practice nurse)*'I think they can be very useful because I think they often just start a conversation. They’re a good opener to ask sometimes difficult questions.'* (P12, Female, GP)*'It’s useful for monitoring patients as well. So even for things like depression and so it’s something that you can work on and you can actually see this is what your score was before, it’s better now.'* (P10, Female, GP)
Divergent views of usefulness of QoL tools	*'They can do in private and then it’s returned almost anonymously sometimes they can be more honest with their answers.'* (P3, Female, GP)*'If you’ve got a patient who wants to give an answer that they think the doctor will want or what they* [the patient] *actually wants to come out of* [the consultation] *at the end … the tool can actually do more harm than good.'* (P16, Male, GP)*'Some of them can be very long winded.'* (P15, Female, GP)*'I find it particularly useful partly because time is very precious in general practice and so anything that allows us to generate structured information that can be tracked over time without picking up a lot of time is helpful*.' (P4, Female, GP)

QoL = quality of life.

**Table 3.** Supporting quotes for the 'WAItE design and content considerations' theme

**Sub-theme**	**Example quotes**
Views of the level of focus on weight in the WAItE tool	*'So I have to say I struggle a little bit with this tool because many of the questions, I think, are so non-specific.'* (P4, Female, GP)*'It could be really useful, but maybe not mentioning weight.'* (P2, Female, GP)
Views on the WAItE questionnaire length	*'I think first of all on a positive it’s short and it’s easy to use, and I think that’s a real benefit to it.'* (P15, Female, GP)*'I definitely thought it was a good length, so I didn’t feel it was too long or too short or anything like that, which is good. I certainly think it’s long enough to get some decent information.'* (P13, Male, GP)
Clarity and relevance of WAItE tool items	*'They* [the questions] *certainly look very relatable to children and young people.'* (P11, Female, GP)*'I thought it* [the WAItE questionnaire] *was quite simple and easy to look at, and I think it would be quite easy for them* [young people] *to understand.'* (P14, Female, Practice nurse)*'They* [the questions] *very much suit that age group.'* (P12, Female, GP)*'I think the questions would have been fine for that young man* [a patient with Down’s syndrome]*.'* (P2, Female, GP)*'”People treat me differently when I go out” — differently to who? Differently to when I stay in, or differently to other people I go out with?'* (P4, Female, GP)*'I mean I don’t know how an 11 year old would … I think it all depends on what their mental status is and how smart they are for them to answer those questions.'* (P10, Female, GP)
Views on the WAItE tool response scale	*'People are more likely to answer questionnaires like that where they’ve got tick-boxes.'* (P18, Female, Practice nurse)*'I also think you’ve got, in terms of “never, almost never, sometimes, often, and always”, that’s good. I like the fact you’ve got ”sometimes” in the middle. A lot of questionnaires don’t have an absolute in the middle.'* (P13, Male, GP)*'Now the questions of "never", "sometimes", "often", and "always", when would you say "sometimes" and when would you say "often"?*' (P10, Female, GP)*'I think if this was to generate a number or for example if somebody ticked "always" for everything, then that certainly could help.'* (P16, Male, GP)

WAItE = Weight-specific Adolescent Instrument for Economic-evaluation.

**Table 4.** Supporting quotes for the 'Perceived challenges, benefits, and methods of implementing the WAItE tool' theme

**Sub-theme**	**Example quotes**
Using the WAItE tool during a clinical consultation	*'A lot of people get the impression that a patient is going to think you’re trying to reduce them to a number by getting them to do a questionnaire. And that puts me off using them quite a bit at the end of the day.'* (P13, Male, GP)*'It makes it a bit more robotic … it can actually take out some of the interpersonal relationship that you have with the patient … actually what you may find if you do it the second way, in open questions, is you get so much information.'* (P16, Male, GP)*'It’s just much more subtle and its questions, I think, are really good … It’s actually opening a line of questioning for the future rather than just are you worried about your weight, would you like to make a change, which is very difficult to move from there.'* (P3, Female, GP)*'And it either works as a complete tick-box so you get a number from it, or as an opening to the consultation and discussion. You can use them either way, so if somebody came back with that I’d say “well, I never get tired and I can keep up, but I feel embarrassed shopping for clothes.” That would be the start of your conversation to intervene.'* (P5, Male, GP)*'We’re not very good at addressing the underlying problems, I think. When we do see children that are overweight we tend to deal with the sore throat or the rash or whatever it is they’ve come in with and forget to deal with it, and I think this* [the WAItE] *is a useful way of addressing it.'* (P12, Female, GP)
Ways to encourage clinicians to use the WAItE tool	*'If it was something that was set in a guideline that this is the kind of gold standard for using then hopefully it would just become common practice.'* (P14, Female, Practice nurse)*'If you got a little flag that says … the little template to say “this person is between those ages, you haven’t got a BMI do you want to measure one?” Or it says “this person is within the age group and their BMI is above let’s say 30, here’s a questionnaire that comes straight up on the screen”, and then you can just hit the button so it’s done in 60 seconds.'* (P5, Male, GP)*'I think the key thing is that there needs to be a what happens at the end of it to be able to refer people on to or something.'* (P19, Female, GP)*'Giving out a questionnaire in my opinion is fine, but what we don’t want is those two actually then have no follow-up.'* (P18, Female, Practice nurse)
Suggestions of future clinical practice using the WAItE tool	*'I guess the other thing is some forms I know these days are getting put into electronic format, like on apps where you can direct children to an app and get them to complete these things … so I guess that would be another thing that young people quite like.'* (P7, Female, GP)*'I was also thinking you might actually want to have a poster or something up in the waiting room with some of these questions.'* (P2, Female, GP)*'Patients, when they check-in, they use an electronic system just to check-in rather than coming to a reception desk … I’m not sure if there’s a way of putting questionnaires on there, is something I’ll have to ask the tech guys, but that might be a way of just doing it when somebody isn’t necessarily attending for a GP appointment.'* (P12, Female, GP)

WAItE = Weight-specific Adolescent Instrument for Economic-evaluation. BMI = body mass index.

### Views about QoL tools in clinical practice

#### Experience of using QoL tools in clinical practice

Participants’ experiences of using QoL tools in clinical practice varied. Some participants used QoL tools frequently in their practice, whereas others did not use them at all. Often participants would use one particular type of condition-specific QoL tool frequently in their practice (for example, a depression and anxiety QoL tool), but were not familiar with and not likely to use many other QoL tools.

#### Advantages and disadvantages of QoL tools

Participants perceived a number of advantages associated with using QoL tools in clinical practice such as the ability to start a conversation and explore a particular topic in detail; the possibility of using QoL tools to monitor improvement (or deterioration) in follow-up consultations; and to facilitate treatment decisions.

There were some divergent views about the accuracy and usefulness of QoL tools. For example, one GP participant believed that QoL tools may help to obtain more accurate information from the patient, while another GP participant believed that patients may not answer QoL tools honestly. In addition, one participant reported a concern about the length of time QoL tools take to complete, whereas another participant believed using QoL tools can save time.

### WAItE design and content considerations

#### The focus on weight in the questionnaire

Some participants perceived the questions in the WAItE questionnaire as not weight-specific enough, whereas other participants felt that the WAItE questionnaire was too weight-specific.

#### The length of the questionnaire

Many participants held positive views regarding the length of the questionnaire and how long it would take to complete it. The questionnaire was perceived to be long enough to capture important information.

#### The clarity and relevance of the questionnaire items

Many participants perceived the questions to be simple and relevant for young people. Some participants perceived the questionnaire to be suitable for children of different ages and for children with developmental difficulties. However, some other participants felt that questions were not as clear as they could be, or that the questions might not be understandable to younger children, or patients with developmental difficulties.

#### The response scale

Some participants had positive views about the response scale, whereas others were uncertain about this aspect of the WAItE tool. Some participants were keen to see an overall score calculated from the responses to the questionnaire items.

### Perceived challenges, benefits, and methods of implementing the WAItE tool

#### Using the WAItE tool during consultations

Concerns were raised that the use of the WAItE tool during a consultation may not be well received by patients and may hinder the rapport between patient and clinician. It was reported that it may depersonalise the communication with a patient by using a questionnaire rather than having a conversation. Another GP reported that the use of the WAItE tool over open questioning may limit the information gained from patients and overlook a GP’s communication skills. However, other interviewees perceived the WAItE tool more positively and anticipated that it could help to start conversations with patients about their weight in a non-judgmental manner. Many of the GPs interviewed considered broaching the topic of weight during a consultation a challenge if a patient attended with a non-weight-related issue, and one GP reported that the WAItE tool could help solve this problem. By acting as a reminder to GPs to discuss a young person’s weight during a consultation when a patient has sought treatment for an unrelated issue, it could help to enhance the consultation by potentially identifying underlying issues rather than just treating symptoms.

#### Encouraging clinicians to use the WAItE questionnaire

Several potential facilitators were identified by the participants to aid the use of the WAItE in primary care. In order to encourage the use of the WAItE tool in routine clinical practice, it was suggested that recommending its use in a clinical guideline and embedding it within GPs’ IT system would be beneficial. Several GPs discussed the importance of having services to refer young people to if the WAItE was to be used in general practice. The completion of the questionnaire alone was not considered sufficient.

#### Suggestions for future use of the WAItE in clinical practice

Clinicians provided suggestions for alternative modes in which the WAItE tool’s future use could be facilitated in clinical practice, such as completing the WAItE tool electronically, having it on a poster in the waiting room, or even adding it to current electronic check-in systems.

## Discussion

### Summary

Clinicians’ use of QoL tools in practice varied. Participants reported that QoL tools are not routinely used in practice with this age group, although, some clinicians had used similar tools to the WAItE tool with patients. Those with experience of using QoL tools in practice reported the benefits of using them to explore a particular topic in detail and of having a structured way to assess patients’ progress at follow-up. On the other hand, some participants believed patients did not like completing tools or questionnaires. In general, participants perceived advantages of the WAItE tool such as starting conversations about weight in a non-judgmental manner. Although, to be truly beneficial, it would be necessary to have effective and stigma-free services available to which patients could be referred. Furthermore, GPs proposed that the WAItE could help address a longstanding challenge of broaching the topic of weight with patients who are overweight when a patient presents with a non-weight-related issue. Disadvantages of the tool were also expressed, such as completing and interpreting the results of the tool within the 10-minute consultation window, and potentially hindering the rapport between patient and clinician.

### Strengths and limitations

The current study provides a novel exploration of the feasibility and acceptability of using a QoL tool specifically designed for young people who are overweight in a primary care setting. The importance of refocusing discussions on QoL to engage children and parents with treatment for obesity has been demonstrated in a UK-based study of childhood obesity,^17^ which demonstrated that perceived improvements in the QoL of children motivated parents to enter their child into treatment. Furthermore, observed benefits in QoL during treatment motivated continued engagement with services.^17^

The current study is novel in its approach of seeking the views of primary healthcare clinicians regarding the use of QoL tools with young people who are overweight and obese. The study explored GPs’ and practice nurses’ views in depth, reaching saturation in the data from participants in primary healthcare settings with varying deprivation. Furthermore, this study explored the use of QoL tools in an English NHS setting, whereas the majority of existing literature has explored QoL tools in a US-only context. However, the English focus prevents the generalisability of the findings to other countries and different healthcare systems.

The majority of the study sample were GPs, which limits the findings to a narrow selection of primary healthcare clinicians. In addition, the interview sample was limited in ethnic diversity with the majority of participants self-reporting as White British. Furthermore, the proportion of patients aged <18 years at the majority of the primary care practices involved in the study were below the UK average. This may have implications for interview participants’ experiences discussing weight with young people.

### Comparison with existing literature

There are a small number of existing tools that can be used to assess the impact of weight status on the QoL on young people such as IWQOL-Kids (Impact of Weight on Quality-of-life-Kids version),^18^ Sizing Me Up,^19^ and YQOL-W (Youth Quality of life Weight).^20^ However, the applicability of these in the context of the UK primary care setting is limited. These tools are lengthy (all three have in excess of 20 questions), which may reduce completion rates and increase the time needed to complete. Furthermore, cultural factors play an important role in perceptions of weight and weight-related health consequences; hence, the generalisability of these tools to the UK population is unclear.^7^

### Implications for research and practice

A future feasibility study will focus on implementing the WAItE tool in GP surgeries. The ultimate goal is the implementation of the WAItE tool within healthcare services and in schools to improve QoL and management of overweight and obesity. Overall the impetus of the implementation of the WAItE tool is to improve clinical practice in the context of weight management for children and young people, and for children and young people to feel listened to in a non-stigmatising way.
